# A randomized controlled trial of supervised group exercise therapy in patients with clinical depressive and anxiety disorders: the challenge of patient compliance

**DOI:** 10.1192/j.eurpsy.2025.352

**Published:** 2025-08-26

**Authors:** Q. Zhai, M. Folkesson, C. Hörnsten, J. Persson, S. Montgomery, Y. Freund-Levi

**Affiliations:** 1 School of Medical Sciences, Örebro University; 2 Department of Psychiatry, Örebro University Hospital; 3 School of Health Sciences, Örebro University, Örebro; 4 Department of Clinical Sciences, Psychiatry, Umeå University, Umeå; 5 School of Behavioural, Social and Legal Sciences, Örebro University, Örebro; 6 Department of Neurobiology, Care Sciences and Society; 7 Clinical Epidemiology Division, Department of Medicine Solna, Karolinska Institutet, Stockholm, Sweden; 8 Department of Epidemiology and Public Health, University College London, London, United Kingdom; 9 Department of Clinical Science and Education, Södersjukhuset, Karolinska Institutet, Stockholm; 10 Department of Geriatrics, Örebro University Hospital, Örebro; 11 Department of Geriatrics, Södertälje Hospital, Södertälje, Sweden

## Abstract

**Introduction:**

Depression and anxiety are global mental health concerns and contribute significantly to the global burden of human disease. Although psychotherapies and antidepressant drugs are effective and commonly used treatments for depression and anxiety, some patients do not achieve full remission of their symptoms and there remains a risk of residual symptoms.

**Objectives:**

To validate effect of supervised group exercise therapy in outpatient treatment of depressive and anxiety disorders.

**Methods:**

A total of 126 individuals were screened for elevated depressive and anxiety symptoms. 86 participants aged between 18 and 65 years (Median=33 years; IQR 15; 62.8 % females) were randomly assigned to exercise group (EX, N=43) or relaxation group (REL, N=43). EX was planned to receive 36 sessions and REL 12 sessions during a 12-week intervention. Depressive symptoms were measured using Montgomery-Åsberg Depression Rating Scale (MADRS and MADRS-S) and anxiety symptoms using Becks Anxiety Inventory (BAI) at pre-, mid- and post-intervention.

The study is registered on ClinicalTrials.gov Register for RCT’s (NCT04714528).

**Results:**

Intention-to-treat analyses showed that both groups improved significantly in depressive and anxiety symptoms (MADRS: EX from 24.5±7.4 to 15.8±8.3 points, REL from 22.8±6.4 to 16.6±7.8 points. MADRS-S: EX from 27.6±7.2 to 19.6±8.1 points, REL from 26.3±7.1 to 18.7±9.5 points. BAI: EX from 25.0±12.2 to 16.4±9.9 points, REL from 24.3±12.4 to 17.0±12.6 points). However, there was no statistically significant difference between the groups in the degree of improvement. In addition, there was a high and differential attrition rate (51.2 % in EX vs 20.9 % in REL, p=0.007). It took longer than expected for the exercise group to finish the intervention (EX 19.2±5.1 weeks vs REL 14.8±4.0 weeks). Attrition analysis showed that individuals that had student as their main occupation were more likely to drop out and participants with sleep medication were more likely to adhere to interventions.

**Image 1:**

**Figure:**
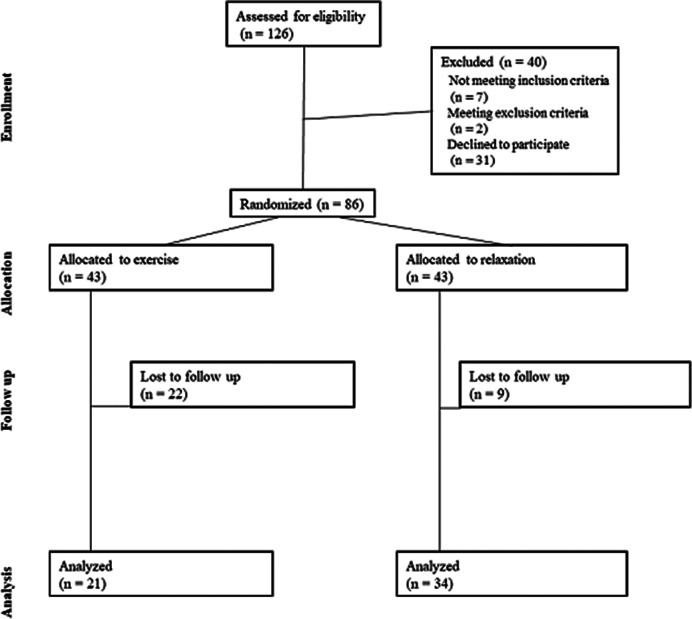

**Image 2:**

**Figure:**
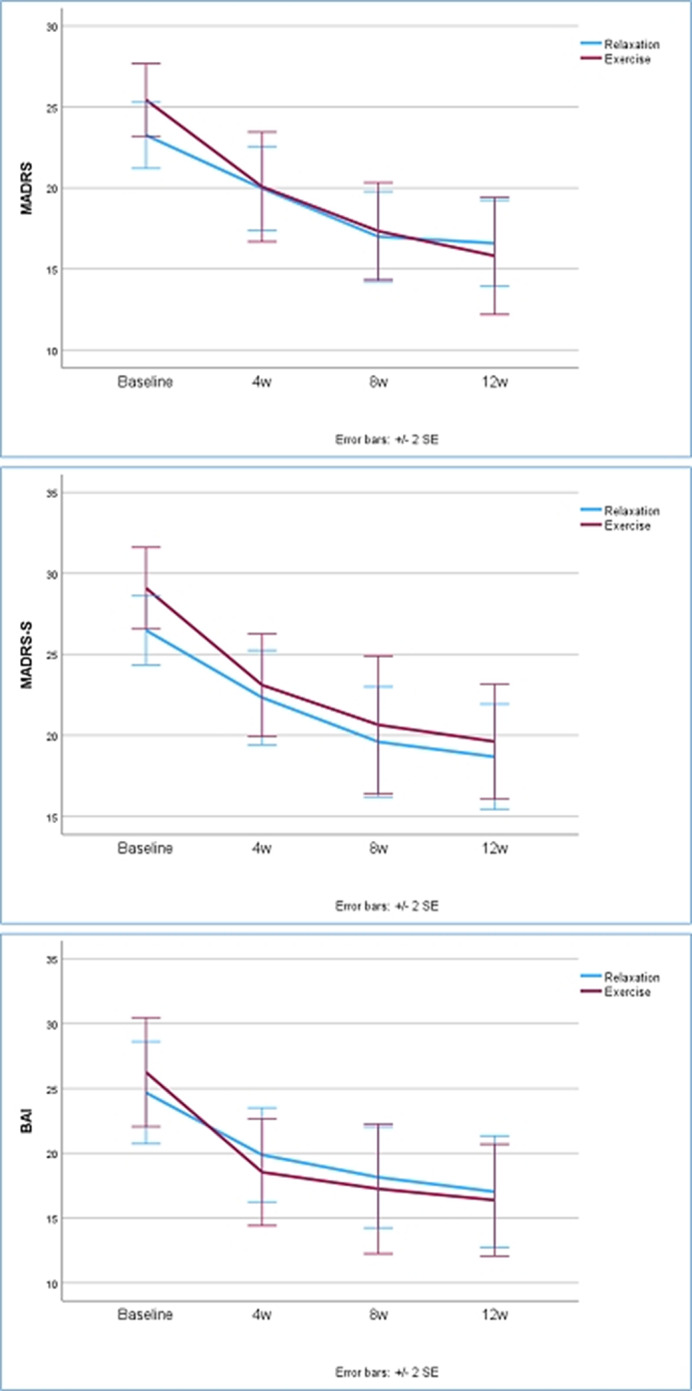

**Conclusions:**

The study reflects the challenges of outpatient, activity-based treatments for mild to moderate depressive and anxiety disorders regarding compliance, despite great efforts were made to keep the participants’ motivation throughout the study. Although both groups improved in depression and anxiety symptoms similarly, due to the lack of control group with no active treatment, it is difficult to assess possible placebo effect from this study. The differential bias from attrition and compliance makes it difficult to draw definitive conclusions.

**Disclosure of Interest:**

None Declared

